# Cerium oxide nanoparticles inhibit lipopolysaccharide induced MAP kinase/NF-kB mediated severe sepsis

**DOI:** 10.1016/j.dib.2015.04.023

**Published:** 2015-05-08

**Authors:** Vellaisamy Selvaraj, Niraj Nepal, Steven Rogers, Nandini D.P.K. Manne, Ravikumar Arvapalli, Kevin M. Rice, Shinichi Asano, Erin Fankenhanel, J.Y. Ma, Tolou Shokuhfar, Mani Maheshwari, Eric R. Blough

**Affiliations:** a Center for Diagnostic Nanosystems, Marshall University, Huntington, WV, USA; b Department of Pharmacology, Physiology and Toxicology, Joan C. Edwards School of Medicine, Marshall University, Huntington, WV, USA; c Department of Cardiology, Joan C. Edwards School of Medicine, Marshall University, Huntington, WV, USA; d Department of Pharmaceutical Sciences and Research, School of Pharmacy, Marshall University, Huntington, WV, USA; e Health Effects Laboratory Division, NIOSH, Morgantown, WV, USA; f Department of Mechanical Engineering and Engineering Mechanics, Michigan Technological University, Houghton, MI, USA

**Keywords:** Sepsis, LPS, Sprague Dawley rat, MTT, Raw 264.7, Cerium oxide nanoparticles

## Abstract

The life threatening disease of sepsis is associated with high mortality. Septic patient survivability with currently available treatments has failed to improve. The purpose of this study was to evaluate whether lipopolysaccharide (LPS) induced sepsis mortality and associated hepatic dysfunction can be prevented by cerium oxide nanoparticles (CeO2NPs) treatment in male Sprague Dawley rats. Here we provide the information about the methods processing of raw data related to our study published in Biomaterials (Selvaraj et al., Biomaterials*,* 2015, In press) and Data in Brief (Selvaraj et al., Data in Brief, 2015, In Press). The data present here provides confirmation of cerium oxide nanoparticle treatments ability to prevent the LPS induced sepsis associated changes in physiological, blood cell count, inflammatory protein and growth factors in vivo. In vitro assays investigation the treated of macrophages cells with different concentrations of cerium oxide nanoparticle demonstrate that concentration of cerium oxide nanoparticles below 1 µg/ml did not significantly influence cell survival as determined by the MTT assay.

Specifications table (please fill in right-hand column of the table below)

**Table:** 

Subject area	Biology
More specific subject area	Nanomedicine
Type of data	Table and figure
How data was acquired	Observational data, MTT assay, complete blood count, cytokine array
Data format	Raw and analyzed
Experimental factors	Rodent based intervention study examining the effects of cerium oxide nanoparticle treatment on LPS induced sepsis.
Experimental features	Balanced design that encompasses both in vivo and in vitro observations
Data source location	Huntington, WV USA
Data accessibility	The data presented in this article and is related to [1,2]

Value of the data

• The data can be referenced by other scientists investigating the effects of LPS on animal behavior and serum cytokine/chemokine levels.
• The data can provide comprehensive analysis of the effect of cerium oxide nanoparticles on LPS-induced sepsis.
• These data provides a more thorough understanding of the hepatic involvement in LPS-induced sepsis.

## 1 Data, experimental design, materials and methods

### 1.1 Experimental design and methods

This data articles contains data related to the research articles entitled “Inhibition of MAP kinase/NF-kB mediated signaling and attenuation of lipopolysaccharide induced severe sepsis by cerium oxide nanoparticles” in Biomaterials [1]. In the present study we have confirmed how the administration of a single dose (0.5 mg/kg) of CeO2NPs intravenously into septic rats prevent the LPS induced sepsis associated physiological, blood cell count and inflammatory protein and growth factors changes. In addition to that we treated the macrophages cells with different concentrations of cerium oxide nanoparticle to determine the range of cytotoxic and non-cytotoxic concentrations. Finally we have hypothesized the potential mechanistic model of LPS induced sepsis. Concentration of cerium oxide nanoparticles below 1 µg/ml did not appears to significantly influence cell survival as determined by the MTT assay. Finally we have showed the mechanistic insight of systemic inflammatory response syndrome (SIRS) of sepsis and effect of CeO_2_ nanoparticles treatment.

#### 1.1.1 Animal preparation and experimental design

Male Sprague-Dawley rats weighing 300–350 g were obtained from Charles River laboratories and housed two per cage using a 12:12- h dark-light cycle at a temperature of 22±2 °C. Animals were provided food and water ad libitum and allowed to acclimate for at least two week prior to any experimentation. Animals were randomly assigned to the following groups: control group (*n*=6) received 1.5 ml of endotoxin free water by i.p., CeO_2_ nanoparticle treated group (*n*=6) received 1.5 ml of endotoxin free water by i.p., and CeO_2_ nanoparticles (0.5 mg/kg) in 200 µl of sterile distilled water via the tail vein. All procedures were performed as outlined in the guide for the care and use of laboratory animals as approved by the council of the American physiological society and the institutional animal use review board of Marshall University.

#### 1.1.2 SDS-PAGE and immunoblotting

Approximately 100 mg of frozen tissue was taken and pulverized in liquid nitrogen and added to 900 µl of T-PER (Pierce, Rockford, IL, USA) containing 1% protease and phosphatase inhibitors (P8340 and P5726, Sigma- Aldrich, St. Louis, MO, USA). Samples were homogenized and centrifuged at 13,000 rpm for 10 min at 4 °C to collect the supernatant. Amount of protein in the samples was estimated through 660 nm assay (Pierce, Rockford, IL, USA) and normalized with T-PER and 4× Laemlli buffer to a final equal concentration across all samples. Equal amount of protein was loaded in 10% PAGEr Gold Precast gel (Lonza, Rockland, ME) and transferred to nitrocellulose membranes using standard protocol. Membranes were block with 5% milk in TBST for 1 h at room temperature, washed thrice with TBST and probed for detection of MyD-88, total and P-p-38 MAPK, total and P- p-ERK 1/2 MAPK (44/42), iNOS, COX-2, HMGB1, Total and cleaved caspase-3, Bax and Bcl-2, GAPDH (Cell Signaling Technology, Danvers, MA). Membranes were incubated with primary antibody overnight at 4 °C, washed with TBST (3×5 min), and incubated with secondary anti-rabbit (Cell Signaling Technology, Danvers, MA) for 1 h at room temperature. Immunoreactive signal was visualized using Supersignal West Pico Chemiluminiscent substrate (Pierce, Rockford, IL, USA) and quantified using Fluorchem 9900 software (Protein Simple, Santa Clara, CA). Protein expression was normalized to glyceraldehyde 3-phosphate dehydrogenase (GAPDH).

#### 1.1.3 TUNEL assay

TUNEL staining was performed on tissue sections (5 µm) of frozen samples, which were dewaxed and rehydrated according to the manufactures recommendation (Roche Applied Science, Indianapolis, IN). Additional experiments were performed in parallel using DNaseI or the omission of labeling reagent was used as positive and negative control. Images were captured using an EVOSfl fluorescence (Fisher Scientific, Pittsburgh, PA, USA) microscope at 200× magnification. TUNEL positive apoptotic nuclei were counted and expressed as the number of TUNEL-positive nuclei per 500 nuclei (TUNEL+/500 nuclei).

#### 1.1.4 Macrophage culture and uptake of CeO_2_ NPs and protective effective of CeO_2_ NPs against LPS

RAW264.7 cells from (ATCC) were grown in 25 cm^2^ cell culture flasks at 30 °C with 5% CO_2_ in DMEM high glucose medium containing 1% Pen/Strep (10,000 U Penicillin and 10 mg Streptomycin/ml) and supplemented with 5% of fetal bovine serum. The cytotoxic and non-cytotoxic concentration of CeO_2_ nanoparticles was determined by MTT assay. The protective effect of CeO_2_ nanoparticles again LPS induced cytotoxicity was measured by MTT assay as follows. RAW 264.7 (1.2×10^5^/ml) were grown in 24 well tissue culture plates until 70–80% confluence and the media was replaced with the fresh medium containing different doses of CeO_2_ NPs (0, 1, 5, 10, 25, 50, 100 and 1000 ng/ml) for 24 h in the absence and presence of LPS and performed MTT assay. To examine if the CeO_2_ nanoparticles exhibit the ability to sequester or neutralize LPS functionality of LPS, different doses of CeO_2_ NPs (0, 1, 5, 10, 25, 50, 100, or 1000 ng/ml) were incubated for 2 h at room temperature in growth medium in the presence or absence of LPS (2 µg/ml). After centrifugation (5000×*g* for 10 min) to pellet any suspended nanoparticles, the now clarified media (500 µl) was added to cultured RAW 264.7 cells for 24 h and the MTT assay performed as detailed previously.

#### 1.1.5 Determination of reactive oxygen species (ROS), mitochondrial membrane potential (Δ*ψm*), nitric oxide (NO) and cytokine levels

Reactive oxygen species (ROS) levels were determined following the addition of 2, 7-dichlorodihydrofluorescein diacetate (DCFH-DA), using the OxiSelectTM kit from Cell Bio Labs (San Diego, CA) as outlined by the manufacturer. The assay employs the cell-permeable fluorogenic probe (DCFH-DC). The DCFH-DC is diffused into cells and is deacctylated by cellular esterases to non-fluorescent DCFH, which is rapidly oxidized to highly fluorescent 2′,7′-dichlorodihydrofluorescein(DCF) by ROS. Briefly, RAW cells were grown in 24-well plates, after the treatment period, cells were washed with cold PBS and incubated in serum-free medium with 100 µl of 1× DCFH-DA reagent and incubated at 37 °C for 1 h. Following this incubation period, cells were washed twice with DPBS and terminated the assay by adding 100 µl of the 2× cell lysis buffer and, the fluorescence of each cell suspension was measured with a fluorometric plate reader (Spectramax, Gemini EM) at 480 nm/530 nm. Observation of dye uptake by cells was determined following imaging using an EVOSfl fluorescence (Fisher Scientific, Pittsburgh, PA, USA) microscope equipped with a GFP filter.

The extent of mitochondrial membrane damage was determined by measuring mitochondrial membrane potential (Δ*ψm*), using the JC 1 dye (Cell Technology, Mountain View, CA). Experimentally treated cells were washed with cold PBS, removed with trypsin and centrifuged at 400×*g* for 10 min. The resulting pellets were then re-suspended in PBS and the cell density adjusted to 1×10^5^ cells/ml. Aliquots (200 µl each) were then centrifuged at 400×*g* for 5 min, and the resulting cell pellets were assayed for mitochondrial membrane damage. In the absence of mitochondrial damage, the JC-1 dye accumulates in the organelle and fluoresces red. Conversely, an inability of the mitochondria to concentrate JC-1 dye results in the accumulation of the dye in the cytoplasm and a green fluorescence. Overall fluorescence was measured with a fluorometric plate reader (Spectramax, Gemini EM) at 535 nm and 600 nm for green and red fluorescence, respectively. Observation of dye uptake by mitochondria was determined following imaging under fluorescence (EVOSfl Model, Fisher Scientific, Pittsburgh, PA, USA).

RAW 264.7 cells were treated with different concentration of CeO_2_ nanoparticles in the presence and absence of LPS (2 mg/ml) for 24 h. Nitrite production in the culture supernatants was assayed using the Griess reaction kit from Cayman Chemical Company (Ann Arbor, Michigan, USA). One hundred microliters was removed from the medium and incubated with an equal volume of Griess reagent for 30 min at room temperature and the absorbance was measured at 540 nm in an ELISA reader (BioTek, Instrument, Inc., Winooski, Vermont, USA) as outlined by the manufacturer. Nitrite concentration was calculated with reference to a standard curve obtained using NaNO_2_.

Cells were cultured for 24 h in the presence of CeO_2_ nanoparticles with and without LPS. Cell culture media was recovered by centrifugation at 400×*g* for 10 min. The concentration of TNF-α, IL-6, and IL-1β in the media was measured by ELISA reagent kits (BD Bioscience, Franklin Lakes, NJ, USA) as detailed by the manufacture. HMGB1 concentration in the media was estimated by ELISA reagent kits (Chondrex Inc, Red mound, WA, USA)

#### 1.1.6 Immunoblotting, electromobility shift assay, and luciferase reporter assay

Cells were washed with cold PBS, collected by scraping and centrifuged at 400×*g* for 10 min. Total cell lysates, cytoplasmic, and nuclear fractions were prepared by cell lytic^TM^ M cell lysis reagent (Sigma) and NE-PER cell lysis buffer (Thermo Scientific, Rockford, IL, USA) as outlined by the manufacturer. Protein content was estimated in triplicate using the Bradford reagent with bovine serum albumin as a standard. Western blot was performed as mentioned elsewhere. Fifty µg of total protein per well was then subjected to electrophoresis and transfer to nitrocellulose Hybond-C membranes (AmershamTM HybondTM) using standard conditions. Membranes were incubated overnight at 4 °C with the appropriate primary antibody iNOS, COX-2, I_k_B-α, and NF-kβ ( Cell Signaling, Dnavers, MA), washed extensively and then incubated for 1 h at room temperature with a horse radish peroxidase labeled anti-rabbit before detection by ECL (Western Blotting Detection Reagent, GE Health Care Amersham, Piscataway, NJ). Immunoreactive signals were quantified by densitometry using Alpha Innotech software (Santa Clara, California). Beta actin immunoreactivity was used for normalization between samples.

The electromobility shift (EMSA) assay was performed using a commercially available kit (Pierce, Rockford, IL, USA) as detailed by the manufacturer Briefly, 5 µg of the nuclear protein extract was used in a binding reaction with 1× binding buffer, 2.5% glycerol, 5 mM MgCl_2_, 50 ng/µl of poly (dI:dC), and 0.05% Nonidet P-40. A double stranded 5′-biotin-NF-kB oligonucleotide probe (consensus sequence 5′-AGTTGAGGGGACTTTCCCAGGC-3′) was added to the reaction at a final concentration of 10 pM/20 µl reaction mix. After 30 min, 5 µl of 5× loading buffer was added to the reaction mix, and the samples were resolved on 6% polyacrylamide gels at 120 V for 55 min using 0.5% TBE before transfer to nylon membrane at 10 V for 70 min using 0.5% TBE. The protein-DNA probe complexes were cross-linked with a UV cross linker. NF-kB specific bands were detected by streptavidin-horseradish peroxidase conjugate using a chemiluminescence nucleic acid detection kit (Thermo scientific, Rockford, IL, USA).

NF-_k_B reporter construct were purchase from Promega (Madison, WI, USA). For the report assay, cells were seeded into 24 well plates at a density of 5×10^5^ cells per well and transiently transfected with 400 ng of luciferase reporter construct and 100 ng of internal control plasmid of the pCMV-β-galactosidase reporter plasmid from Clontech (Mountain view, CA, USA) using lipofectamin TM200 reagent according to the manufacture’s procedure (In vitrogen, Carlsbad, CA, USA). Twenty four hours after transfection, the cells were treated with fresh medium containing LPS and LPS +CeO_2_ nanoparticles for further 24 h. After 24 h, the luciferase activities were measured in cell lysates using luciferase and x-gal reagents following manufacture’s instruction (Promega, Madison, WI, USA and Clone tech, Mountain view, CA, USA).

## 2 Results for the present study

### 2.1 Physiological behavior and morphological changes

In Table 1 physiological behavior and morphological changes among different experimental groups is represented. In control animals, there were no changes in appearance, breathing rate, weight change, and animal behavior and, provoked reaction. Whereas LPS induced septic animals showed changes in animal morphology, roughened fur, fast breathing rate, slow moment and no flight reaction at all. In contrast, the behavior and morphological changes of animals from LPS+ CeO_2_ nanoparticles group was similar to that from the control animals.

### 2.2 Complete blood cell counts

Table 2 exhibits the result of blood cells count among different experimental groups. Compared to control animals, the septic animal’s white blood cells count at 6 h and total and percent (%) lymphocytic count both at 6 and 24 h time points dropped significantly compared to control, whereas percent (%) granulocyte count at both time points increased significantly. Nanoparticle treatment increased the total and percent (%) lymphocyte count and decreased the percent (%) granulocyte count significantly.

### 2.3 Inflammatory protein and growth factors

Table 3 demonstrates the results of inflammatory protein and growth factors among the different experimental groups. Compared to the data obtained from control animals, septic animals appears in increased the levels of inflammatory proteins and growth hormones such stem cell factor, myoglobin, CD-40 ligand, fibrinogen, growth hormone, hepataglobin, leptin and IP-10 both at 6 and 24 h time points. Nanoparticles treatment decreased LPS-induced myoglobin at 6 h and 24 h and, the stem cell factor, CD-40, fibrinogen at both time points.

### 2.4 Cytotoxicity

**Fig. 1:**
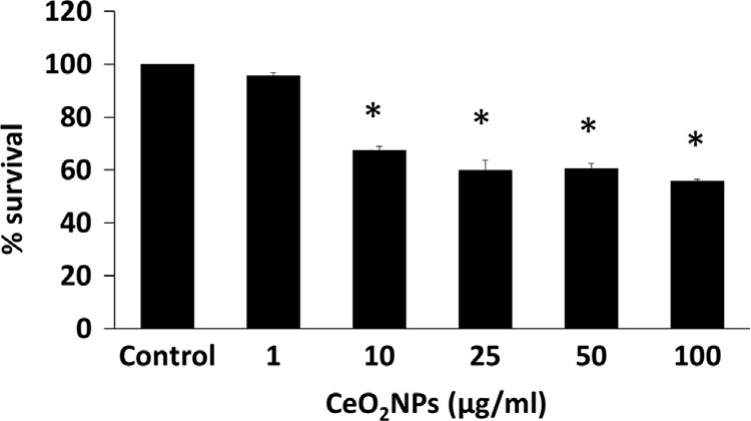
Determination of cytotoxic and non-cytotoxic concentration of CeO_2_ NPs. ^*^P<0.05 compared to control group.

Fig. 1 reveals the cytotoxic and non-cytotoxic concentration determination in RAW cells. Concentration of cerium oxide nanoparticles below 1 µg/ml did not appears to significantly influence cell survival as determined by the MTT assay.

### 2.5 Potential mechanistic model

**Fig. 2:**
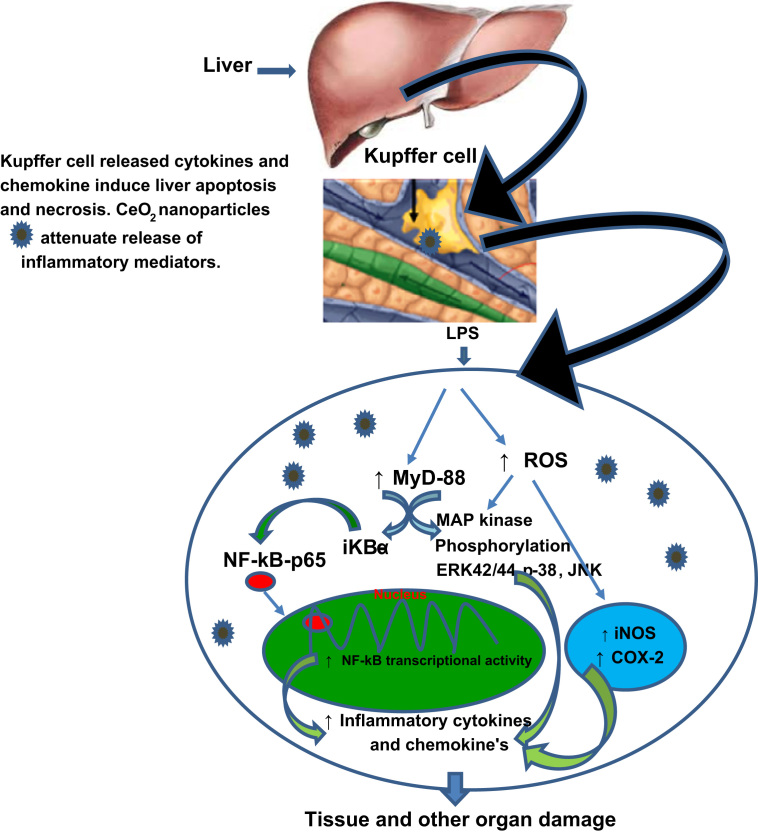
shows the mechanistic insight of systemic inflammatory response syndrome (SIRS) of sepsis and effect of CeO_2_ nanoparticles treatment.

Fig. 2 shows the mechanistic insight of systemic inflammatory response syndrome (SIRS) of sepsis and effect of CeO_2_ nanoparticles treatment. The liver plays a central role in lipopolysaccharide (LPS) induced sepsis. LPS from the circulation may mat illicit a response from the liver causing a release of cytokines and reactive oxygen mediators. Kupffer cells (macrophages) represent the main cellular mediators of the effects of LPS in the liver [3]. LPS interacts with Toll like receptor 4 (TLR-4) which is present on cell membrane of macrophage. As the result of interaction, myeloid differentiation protein 88 (MyD88) initiates early activation of NF-kB. Upon activation TLR4 with LPS, MyD 88 activates Ikβ kinases, leading to phosphorylation and degradation of IkB and release and translocation of NF-kB to the nucleus as well as induced expression of proinflammatory gene and expression of pro-inflammatory cytokines and chemokines, which causes for liver and other organ damage [4]. At the same time, LPS leads to secrete elevated reactive oxygen species (ROS) which trigger mitogen-activated protein kinase (MAPK) signaling pathway. Activated MAPK kinase plays a crucial role in the release of specific cytokines and chemokines which causes for liver and other organ damage [5].

## 3 Conflict of interests

The authors declare that they have no competing interests.

## Figures and Tables

**Table 1:** Effect of CeO_2_ nanoparticles on LPS induced septic symptoms (physical behavior and morphological changes).

Physical behavior	External symptoms/sign	Score	Control & CeO_2_										LPS					
			0 h	6 h	12 h	24 h	48 h	72 h	96 h	120 h	144 h	168 h	0 h	6 h	12 h	24 h	48 h	72 h
Appearance	Normal, Smooth Fur	0	0	0	0	0	0	0	0	0	0	0	0					0
	Roughened Fur	1												1	1	1	1	
	Wet Fur	2																
	Mucous eyes	3																
Breathing	Normal	0	0	0	0	0	0	0	0	0	0	0	0					0
	Fast	1												1	1	1	1	
	Slow	2																
	Weak and intermittent	3																
Weight changes	j 5%	0	0										0					
	j 15%	1		1														
	j 20 %	2			2	2	2	2	2	2	2	2		2	2	2	2	2
	Greater than j 20%	3																
Behavior	Normal, agile, prying	0	0	0	0	0	0	0	0	0	0	0	0					0
	Slow movement, sitting position	1												1	1	1	1	
	Dull, Sloughed, tottering movements	2																
	Lateral position	3																
Provoked reaction	Escape reaction when cage is opened	0	0	0	0	0	0	0	0	0	0	0	0					
	Fight when approached by hand	1																
	Flight when touched	2																
	No flight reaction at all	3												3	3	3	3	3

Physical behavior	External symptoms/sign	Score	LPS				LPS+CeO_2_									
			96 h	126 h	144 h	168 h	0 h	6 h	12 h	24 h	48 h	72 h	96 h	120 h	144 h	168 h
Appearance	Normal, Smooth Fur	0	0	0	0	0	0	1			0	0	0	0	0	0
	Roughened Fur	1							1	1						
	Wet Fur	2														
	Mucous eyes	3														
Breathing	Normal	0	0	0	0	0	0	0			0	0	0	0	0	0
	Fast	1							1	1						
	Slow	2														
	Weak and intermittent	3														
Weight changes	j 5%	0														
	j 15%	1														
	j 20 %	2	2	2	2	2	0	2	2	2	2	2	2	2	2	2
	Greater than j 20%	3														
Behavior	Normal, agile, prying	0	0	0	0	0	0				0	0	0	0	0	0
	Slow movement, sitting position	1						1	1	1						
	Dull, Sloughed, tottering movements	2														
	Lateral position	3														
Provoked reaction	Escape reaction when cage is opened	0		0	0	0	0				0	0	0	0	0	0
	Fight when approached by hand	1	1					1	2	2						
	Flight when touched	2														
	No flight reaction at all	3														

Result: Control (*n*=6), CeO_2_ (*n*=6), LPS (*n*=12) and LPS+ CeO_2_ (*n*=12).

**Table 2:** Effect of CeO_2_ nanoparticles on LPS induced changes in blood cells.

	**Whole blood cells counts**							
	**6** **h**				**24** **h**			
	**Control**	**CeO**_**2**_	**Sepsis**	**Sepsis+CeO**_**2**_	**Control**	**CeO**_**2**_	**Sepsis**	**Sepsis+CeO**_**2**_
**WBC (109/l)**	3.03±0.36	2.81±0.38	1.39±0.27*	1.27±0.44	3.4±0.74	2.99±0.46	2.835±0.39	2.87±0.45
**Lymphocytes (10**^**9**^**/l)**	2.00±0.20	0.82±0.20*	0.37±0.05*	0.82±0.11*#	2.40±0.55	1.96±0.12	0.54±0.54*	0.43±0.44
**Monocytes (10**^**9**^**/l)**	0.08±0.04	0.11±0.06	0.07±0.03	0.03±0.00	0.18±0.05	0.155±0.04	0.17±0.05	0.22±0.05
**Granulocytes (10**^**9**^**/l)**	1.03±0.18	1.87±0.32*	0.946±0.12	0.616±0.34	0.815±0.16	0.875±0.34	2.13±0.29*	2.21±0.38
**Lymphocytes (%)**	65.80±4.11	50.85±6.69	27.88±2.07*	62.96±8.53*#	70.00±3.79	68.75±4.75	19.41±1.79*	15.80±1.15
**Monocytes (%)**	2.55±1.20	1.95±1.71	5.166±1.61	2.98±0.75	5.18±0.74	6.58±2.01	6.41±1.85	6.71±1.72
**Granulocytes (%)**	31.56±3.55	60.70±6.13^‡^	66.9±1.19*	29.9±9.24*#	24.81±4.16	24.64±6.30	74.16±1.81*	76.18±2.04

⁎ *P*<0.05 compared to control group.

⁎# *P*<0.05 compared to sepsis group. (*n*=6/group).

**Table 3:** Effect of CeO_2_ nanoparticles on LPS induced inflammatory proteins.

Analyte	Serum							
	6 h				24 h			
	Control	CeO2	Sepsis	Sepsis+CeO_2_	Control	CeO2	Sepsis	Sepsis+CeO_2_
Stem cell factor (pg/ml)	545±25	616±38	1540±150*	1636±131	655±29	666±11	1193±37*	878±58*#
Myoglobin (ng/ml)	1540±20	738±43*	2080±141*	1170±45*#	702±45	1710±10*	2370±80*	1816±96*#
CD-40 ligand (pg/ml)	428±0	428±0	964±89*	749±80	431±3	428±0	1386±96*	1094±95*#
Fibrinogen (µg/ml)	510±0	510±0	656±47*	540±24	510±0	510±0	1223±61*	901±19*#
Growth hormone (ng/ml)	55666±2603	51000±22143	64666±1333	56000±2516*#	56000±1154	37333±1452*	19666±666*	56333±881*#
Heptaglobin (µg/ml)	635±21	203±2*	222±2*	259±4*#	801±21	607±15*	1093±48*	1326±32
Leptin (ng/ml)	360±26	446±32	1366±33*	1300±57	410±8	406±31	1400±57*	1433±33
IP 10 (pg/ml)	91±0	91±0	1686±18*	1883±69	91±0	91±0	337±11*	363±10

⁎ *P*<0.05 compared to control group.

⁎# *P*<0.05 compared to sepsis group. (*n*=6/group).
